# Lessons From Two Cases of Life‐Threatening Hemorrhage Which Occurred Immediately Before Tooth Extraction in Elderly Cancer Patients With Diabetes

**DOI:** 10.1002/ccr3.72597

**Published:** 2026-04-29

**Authors:** Sumitaka Hagiwara, Reika Hasegawa, Shigeru Kusumoto, Shintaro Beppu, Nobuhiro Hanai

**Affiliations:** ^1^ Department of Dentistry Aichi Cancer Center Hospital Nagoya Aichi Japan; ^2^ Department of Head and Neck Surgery Aichi Cancer Center Hospital Nagoya Aichi Japan; ^3^ Department of Oral and Maxillofacial Surgery Tono Chubu Medical Center Toki Gifu Japan; ^4^ Department of Hematology and Cell Therapy Aichi Cancer Center Hospital Nagoya Aichi Japan

**Keywords:** dental procedure, diabetes, elderly cancer patient, family involvement

## Abstract

Tooth extraction is often required before chemoradiotherapy in patients with cancer. We herein report two cases of elderly cancer patients who experienced life‐threatening hemorrhage immediately before scheduled extractions. Case 1 involved a 79‐year‐old man who underwent chemotherapy for malignant lymphoma. The patient had a history of diabetes mellitus and cerebral infarction. The night before his scheduled tooth extraction, he suffered intracerebral hemorrhaging and was emergently transported to a neighboring general hospital. Case 2 involved a 72‐year‐old man with oropharyngeal cancer who had completed induction chemotherapy. This patient was diabetic, living alone, and failed to appear on the day of scheduled tooth extraction. His sister, living in a neighboring town, found him in an abnormal condition. He was emergently transported to our hospital and diagnosed with gastrointestinal bleeding. These cases highlight the fragility of elderly patients with diabetes undergoing cancer treatment and the importance of perioperative interdisciplinary coordination, including family involvement.

## Key Clinical Message

Dental professionals should be aware that even routine procedures, such as extraction, can cause life‐threatening events due to the systemic instability of elderly cancer patients with diabetes. Interdisciplinary coordination, including family involvement, is essential to prevent fatal outcomes during dental care in this vulnerable population.

## 1 Introduction

Elderly patients require special consideration for dental treatment because of their age‐related frailty and comorbidities [1]. Systemic instability may decrease tolerance to dental procedures, and deterioration of comorbidities may lead to the disturbance of scheduled treatment. In particular, invasive treatments, such as tooth extraction, can be associated with sudden changes in general health status. These circumstances underscore the critical importance of a thorough preoperative assessment and close medical management in geriatric dental procedures.

When cancer treatment is planned, it is recommended to perform any necessary dental work as soon as possible and to provide either preventive or proactive dental care to avoid long‐term complications [2, 3]. Patients currently receiving chemotherapy without radiation of the head and neck region may undergo routine dental procedures [4]. However, for elderly cancer patients, it is necessary to evaluate the patient's overall condition particularly carefully to ensure safe dental procedures due to the risks associated with their physical vulnerability.

In our institution, we routinely perform removal of dental infections in patients with cancer, and the age group of patients undergoing cancer treatment has increased over the past decade. We herein report two elderly patients with diabetes who were receiving chemotherapy and developed life‐threatening hemorrhagic events immediately before scheduled dental extraction. This clinical procedure, which is easily planned by an oral surgeon, may carry a potential risk of emergency even during the preoperative period.

## 2 Case Series

### 2.1 Case 1

A 79‐year‐old man presented to our department in January 2025 for an initial consultation with pain and swelling in the left mandibular region. He had a medical history of hypertension, diabetes, and cerebral infarction, and was receiving antiplatelet therapy with aspirin (Bayaspirin). His alcohol consumption was 700 mL of beer per day (28 g/day), and his smoking history was 20 cigarettes per day for 20 years. He lived with his wife and maintained independence in activities of daily living (ADLs). The patient was diagnosed with peripheral T‐cell lymphoma and received 5 cycles of chemotherapy (BV‐CHP regimen: Brentuximab vedotin [1.8 mg/kg, Day 1], cyclophosphamide [600 mg/m^2^, Day 1], doxorubicin [40 mg/m^2^, Day 1], and prednisolone [100 mg/body, Days 1–5]) at the Department of Hematology and Cell Therapy in our hospital, beginning 14 months prior to his initial consultation at our department. However, owing to disease recurrence, the treatment was switched to oral valemetostat (200 mg/day).

#### 2.1.1 Diagnosis

**FIGURE 1:**
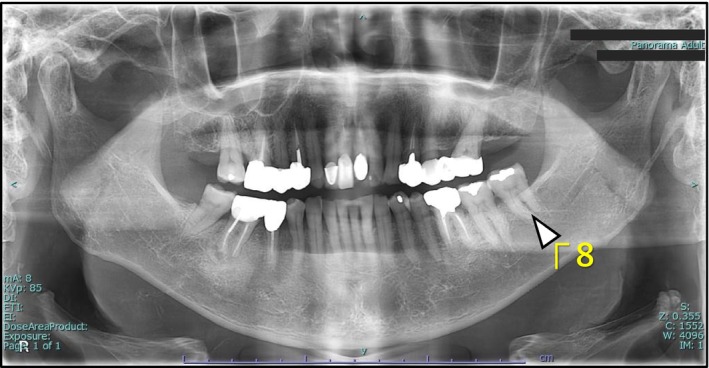
The preoperative orthopantomogram indicates the erupted third molar in the left mandible which was the cause of pericoronitis (white arrow).

He was diagnosed with pericoronitis of the left lower third molar (Figure 1), and antibiotic therapy was initiated.

#### 2.1.2 Treatment

Conservative therapy for pericoronitis was administered for 3 months, but the symptoms recurred and the inflammation extended to the root of the adjacent second molar. After a thorough discussion with the attending physician, it was decided to proceed with inpatient extraction of the left lower second and third molars while continuing antithrombotic therapy with aspirin. The patient also visited our hospital 6 days before the scheduled tooth extraction date to check for inflammation around the tooth to be extracted and to undergo blood tests to confirm his condition. A preoperative blood examination indicated slight anemia and hyperglycemia, with hemoglobin and blood glucose values of 9.7 g/dL and 160 mg/dL, respectively.

#### 2.1.3 Outcome

The night before admission, the patient's condition suddenly deteriorated. Following an emergency call made by a family member, the patient was transported to a neighboring general hospital, where he was diagnosed with an intracerebral hemorrhage (ICH) and received intensive care. The patient was no longer able to continue the treatment for lymphoma and thereafter discontinued the follow‐up at our hospital.

### 2.2 Case 2

A 72‐year‐old man was referred to our department for oral screening before the initiation of definitive chemoradiotherapy and was initially seen in June 2025. He had a medical history of rectal cancer, colon cancer, ossification of the posterior longitudinal ligament, gastric ulcer, and diabetes. His alcohol consumption was 300 mL of sake, 6 days per week (approximately 31 g/day), and his smoking history was 10 cigarettes per day for 51 years. He was living alone but not certified for long‐term care. The patient had been diagnosed with right oropharyngeal squamous cell carcinoma and underwent tracheotomy in March of 2025 at the Department of Head and Neck Surgery in our hospital. Beginning in April 2025, he received three‐cycle induction chemotherapy (PCE regimen: paclitaxel [80 mg/m^2^, Days 1 and 8], carboplatin [AUC; 2.5, Days 1 and 8], and cetuximab [400 mg/m^2^, Day 1, 250 mg/m^2^ on Days 8 and 15]).

#### 2.2.1 Diagnosis

**FIGURE 2:**
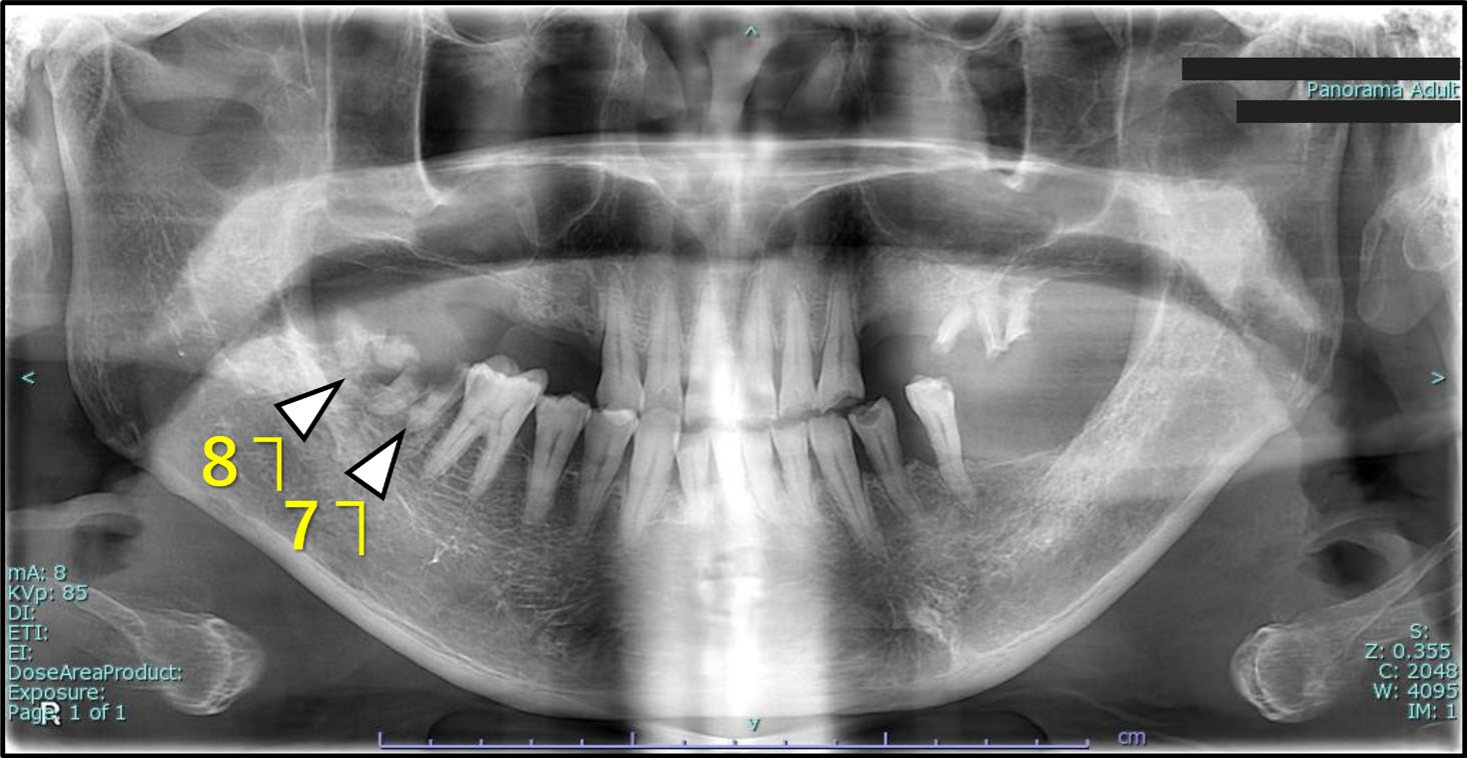
The preoperative orthopantomogram indicates the disrupted second and third molars in the right mandible which were the cause of the apical periodontitis (white arrows).

The right lower second and third molars were diagnosed with apical periodontitis (Figure 2) and planned for extraction as an outpatient procedure the following day.

#### 2.2.2 Treatment

He had no history of taking any nonsteroidal anti‐inflammatory drugs (NSAIDs). A preoperative blood examination revealed anemia and hyperglycemia, with hemoglobin and blood glucose values of 8.3 g/dL and 157 mg/dL, respectively.

#### 2.2.3 Outcome

**FIGURE 3:**
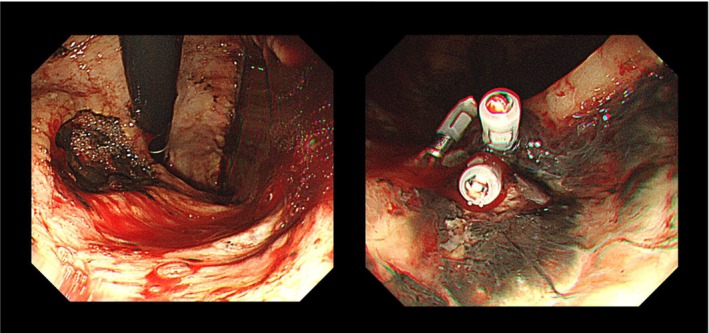
The image of emergency endoscopic hemostasis for gastric bleeding. The left and right panels show the findings before and after hemostasis, respectively.

He failed to appear on the day of scheduled tooth extraction and was not in contact. Since he remained unreachable the next morning, his sister living in a neighboring town was promptly asked to check on him. She found him at home in an abnormal general condition, pale and immobile. He was urgently transported to our hospital, where he was diagnosed with gastrointestinal bleeding secondary to a gastric ulcer. A blood examination revealed a red blood cell count of 131 × 10^4^/μL and hemoglobin value of 3.7 g/dL, and transfusion and emergency endoscopic hemostasis were performed (Figure 3). According to the patient's comment, he had been feeling unwell since the night before the procedure was to be performed and had been unable to leave his house on the day of the procedure. After confirming clinical stability for 2 weeks, tooth extraction was performed. One week later, the wound healed well and definitive radiotherapy without chemotherapy was initiated.

## 3 Discussion

Over the past decade, advances in oral anticancer agents and diversification of chemotherapy regimens have led to an increasing number of patients receiving outpatient cancer treatment. Consequently, there are also more opportunities for outpatients with cancer to undergo invasive dental procedures at the request of the patient's primary physician. Anticancer drugs exert a variety of systemic effects, and safe dental management requires closer communication with the primary physician than ever before. Although coordination with the primary physician was maintained in the two cases presented in this report, cerebral and gastric hemorrhaging occurred immediately before the scheduled tooth extraction. If the tooth extraction had been scheduled 1 day earlier, it is highly likely that an unexpected acute event would have occurred during or after the extraction. In retrospect, it is conceivable that invasive dental procedures could have caused such life‐threatening complications [5]. The occurrence of such events, despite medical collaboration within the same institution, may represent a blind spot in interdisciplinary medical care and a potential risk specific to elderly cancer patients.

Wongiam et al. reviewed the components of a comprehensive geriatric assessment (CGA) required for oral care in elderly patients from the perspectives of multidisciplinary experts [6]. They identified the following assessment items that are considered important when planning invasive dental treatments such as tooth extraction: (1) the assessment of the general condition and risk of comorbid diseases, as to evaluate the risks associated with tooth extraction, it is essential to understand past medical history and chronic diseases (e.g., heart disease and diabetes); (2) the assessment of the ADL using the Barthel Index, as the level of independence in daily living activities (eating, moving, toileting, etc.) provides insight into elderly patients' ability to manage post‐treatment care and recovery effectively; (3) the assessment of nutritional status using the Mini Nutritional Assessment (MNA), as malnutrition increases the risk of delayed wound healing and infection; (4) the assessment of the cognitive function using Mini‐Cog, a simple screening tool that helps confirm the patient's understanding of the treatment and their capacity for self‐care. As demonstrated in this paper, the condition of elderly patient may suddenly deteriorate immediately before invasive dental procedures. Therefore, it is important to ensure that family members or caregivers promptly respond to changes in elderly patients. In other words, it is essential to understand a patient's living environment and social background when planning treatment.

A common feature in both of the present cases was the presence of diabetes, in addition to ongoing cancer treatment. Diabetes causes vascular disorders, such as arterial sclerosis, which can increase the risk of bleeding. Several anticancer agents used in both cases might have caused tissue fragility or mucositis, although each agent is thought to have a low risk of hemorrhage. In Case 1, the BV‐CHP regimen including prednisolone was discontinued more than 1 year previously. In Case 2, dexamethasone was only used for the premedication of the PCE regimen. Therefore, it can be argued that diabetes indirectly elevated the risk of these hemorrhages. Boulanger et al. conducted a meta‐analysis of studies between 1980 and 2014 to examine the effect of diabetes on the occurrence of ICH [7]. In 19 case–control studies involving 3397 ICH patients and 5747 non‐ICH patients, the odds ratio (OR) was 1.23 (95% confidence interval [CI]: 1.04–1.45), which suggested an increased risk of ICH in diabetic patients. In Case 1 described in this paper, it is evident that both diabetes and antithrombotic therapy with aspirin for cerebral infarction triggered ICH. According to current clinical recommendations, simple tooth extraction in patients receiving antithrombotic therapy should be performed without discontinuation of oral antithrombotic agents [8, 9]. It was assumed that aspirin had likely led to the onset of ICH. However, there is also a possibility that ICH could have occurred during the surgical procedure, highlighting the challenges in managing patients with diabetes. In Case 2 described in this study, diabetes was also associated with gastric ulcer. Wei et al. elucidated the relationship between diabetes and the risk of peptic ulcer bleeding (PUB) [10]. Nineteen high‐quality studies were included in their analysis. In a meta‐analysis of morbidity in primary PUB, a random‐effects model showed an OR of 1.433 (95% CI: 1.280–1.604) for the incidence of PUB in diabetic patients compared to nondiabetic patients. In addition, a fixed‐effects model revealed higher 30 day mortality in diabetic patients with PUB (OR: 1.442, 95% CI: 1.245–1.671) than in those without diabetes. Both cases emphasize the importance of careful perioperative management in patients with diabetes.

Another significant implication drawn from these two cases is the critical importance of an effective response from family members or relatives when a patient's physical condition suddenly deteriorates. In Japan, the aging of society has led to an increasing number of elderly individuals living alone, and often managing various health concerns. While advanced age is a known risk factor for health decline due to frailty and other underlying conditions, elderly patients undergoing cancer treatment are particularly vulnerable to sudden health changes caused by the physical burden of therapy. In Case 1, it is possible that the stress of the upcoming tooth extraction contributed to the physiological breakdown. The psychological stress of upcoming surgery is a known factor for blood pressure spikes in elderly patients, and anxiety management is included as part of the recommended protocol [11]. In Case 2, the patient failed to attend the scheduled tooth extraction without prior notice. Concerned about his safety, we contacted his older sister living in a neighboring city, who promptly consulted his residence manager and visited him. This quick intervention plays a critical role in saving lives. Standard referral systems and preoperative evaluations may not anticipate rapidly evolving systemic crises, particularly in elderly patients living alone. In daily clinical practice, it is essential for dental professionals to ascertain the living arrangements of their elderly patients and ensure that an adequate support system is in place to respond appropriately to any sudden deterioration in health status.

## 4 Conclusion

To provide dental treatment to elderly cancer patients with diabetes, it is necessary to have comprehensive medical knowledge of systemic management. Furthermore, to develop a system that can promptly detect changes in the patient's general condition, interdisciplinary coordination, including family involvement, is essential in the period leading up to the procedure.

## Author Contributions

**Sumitaka Hagiwara:** conceptualization, data curation, investigation, writing – original draft, writing – review and editing. **Reika Hasegawa:** writing – review and editing. **Shigeru Kusumoto:** writing – review and editing. **Shintaro Beppu:** writing – review and editing. **Nobuhiro Hanai:** supervision, writing – review and editing.

## Funding

This research did not receive any specific grants from public funding agencies.

## Ethics Statement

This case series was conducted after obtaining approval from the ethics committee of the author's hospital (IR071509).

## Consent

Written informed consent for publication was obtained from one patient. The other patient had previously signed a general consent form at our hospital, which included permission for the use of anonymized clinical data for academic purposes.

## Conflicts of Interest

The authors declare no conflicts of interest.

## Data Availability

All data generated during this study are included in this article.
